# P002: The analysis and impact of three successive intervention programmes directed to reduce central line associated blood stream infections over a four year period in a tertiary care hospital in India

**DOI:** 10.1186/2047-2994-2-S1-P2

**Published:** 2013-06-20

**Authors:** N Jaggi, P Nirwan, E Naryana, KP Kaur

**Affiliations:** 1 Infection control, Artemis Health Insitute, Gurgaon, India

## Introduction

Central line associated blood stream infections (CLABSI) in the ICU are associated with significant morbidity, mortality and costs. Supervision programmes have shown to decrease CLABSI rates but repeated interventions are required.

## Objectives

The objectives of this study were to try to bring CLABSI rates to zero and to promote a culture of safety in the organization.

## Methods

**Setting:** 300-bed tertiary care private hospital. **Study Period:** 4 years (Jan 2009 to Dec 2012)

**Baseline Period:** CLABSI cases were tracked/month from Jan 2009 to June 2009.

♣ **Phase I (July 2009-Jun 2010):** The bundle components for the prevention of CLABSIwere introduced.

♣ **Phase II (Jul 2010-Dec 2011):** This involved introduction of an active, dedicated & trained central line team, dedicated central line trolley, “Scrub the Hub” campaign and involvement of senior doctors and management.

♣ **Phase III (Jan 2012-Dec 2012):** Aim was to reduce CLABSI to zero.

**Outcome measurement** in terms of reduction in CLABSI rates in the pre-and post-intervention phases was done. The results were statistically analyzed by regression analysis and probability assays.

## Results

- The mean CLABSI rate was 2.59 (Range: 0-9.16) infections/1000 catheter days in 4 year period. Mean CLABSI rate in the baseline period: 5.18.

- 21.9% and 82.5% increase was observed in hand hygiene and daily review of line necessity respectively in Phase II as compared to Phase I.

- A significant decrease observed in CLABSI rates after the three supervision programs as 15.3% in Phase I (4.39 p>0.05), 54.9% in Phase II (1.98 p<0.05) and 58.6% in Phase III (0.82 p<0.05).

- Zero CLABSI was observed for three quarters in Phase III. Overall 84% decrease was observed in CLABSI rates in the entire study period.

## Conclusion

- Repeated multimodal supervision programs promoting a culture of safety and zero tolerance effectively reduce CLABSI rates with dedicated central-line teams and involvement of senior management and doctors acting as boosters. A zero tolerance approach resulted in the achievement of a CLABSI free period for three quarters.

## Disclosure of interest

None declared

